# Supplementary material for: Leveraging technology to probe mechanisms of psychopathology: A proof of concept study of inhibitory control
Source: PLoS One. 2025 Jun 6;20(6):e0319004. doi: 10.1371/journal.pone.0319004 (PMC12253936; doi:10.1371/journal.pone.0319004)
Supplement: S1 File — (PDF) [file pone.0319004.s001.pdf]

Leveraging technology to probe mechanisms of psychopathology:
A proof of concept study of inhibitory control

Supplementary Materials

Text S1. Excerpt from Cardinale et al 2021 PLOS One describing the in-laboratory measures of inhibitory control

Antisaccade task. The antisaccade task measures motor inhibition in the context of automatic visual saccades towards novel stimuli. Using a mixed event version of the task, participants are instructed to engage in either prosaccade trial, in which they are instructed to direct their eye gaze towards a visual target, or an antisaccade trial, in which they are instructed to direct their eye gaze in the direction *opposite* a visual target. The order of prosaccade and antisaccade trials are fully randomized within each block. Each trial begins with a preparatory period during which participants are presented with either a green or red instructional fixation cross for either 2 or 6 seconds. The color of the cross indicated the next trial is either a prosaccade (green) or antisaccade (red) trail. This preparatory period was followed by a 200 ms blank screen. Next, during both the prosaccade and antisaccade trials, the response period consisted of a yellow visual target presented for 1 second in a pseudorandomized location 630 pixels or 315 pixels to the left or right of the center of the screen. The number of trials with the visual target in each location is equal across prossacade and antisaccade trials. The testing session consists first of a practice block. After completing the practice, participants completed 3 experimental blocks each with 16 antisaccade and 16 prosaccade trials. The EyeLink 1000 Plus eye tracking system was used to collect and process eye gaze data. For each participant the percentage of correct antisaccade trials was computed as a measure of successful motor inhibition.

AX Continuous Performance Task (AXCPT). The AXCPT is a type of continuous performance task in which children are continuously presented with a series of letters and instructed to press a button in response to each presented letter. Children are instructed to press buttons depending on the sequence of letters presented. Specifically, when a child sees a letter pair A followed by X they are instructed to press 2 in response to the A and 3 in response to the X. For all other letter pairs, the child is instructed to press 2 for both letters. Critically, the majority of the time an X appears on the screen, it is proceeded by an A, meaning that pressing 3 in response to the X quickly becomes a learned prepotent response. Trials can be categorized based on the letter pairings, with trials that contain an A cue with an X probe categorized as "AX" trials and trials containing a non-A cue with an X probe categorized as "BX" trials. In these BX trials, participants must inhibit the prepotent response of pressing 3 and instead press 2 in response to the X. Following a practice phase, during which children receive feedback based on their responses, participants completed three experimental blocks, during which participants completed a total of 150 trials. All trials consist of the following sequence of events: 200-500 ms jittered fixation, 500 ms presentation of the cue, 1000 ms fixation screen, 3900 ms blank screen, 500 ms probe, and a 1700 ms fixation. For each participant, d' context (% correct AX trials—% incorrect BX trials) was calculated as a measure of inhibitory control.

Flanker task. The flanker task letter is a well-established task measuring interference effects on cognitive control. In this task, participants are asked to inhibit interfering task irrelevant information in order to engage in the task goal. Participants are instructed to press the left or right arrow button to indicate the direction of the central arrow in series of five side-by-side arrows centered on the screen. The trial terminates upon response and participants are instructed to respond as quickly as they can. If participants do not respond, the response window closed after 2 seconds. All flanker trials were preceded by a 300ms fixation. Following the participants response, a blank screen was presented for a pseudorandom duration (1000ms – 1500ms). Trials are categorized as either congruent or incongruent trials based on the direction of the flanking arrows. Congruent trials correspond to trials where the flanking arrows all point in the same direction as the central arrow. The congruency of the visual stimuli therefore facilitates the correct motor response. In contrast, incongruent trials correspond to trials where the flanking arrows all point in the opposite direction of the central arrow. The incongruency of the visual stimuli therefore interferes with the execution of the correct motor response. Participants first completed a practice block in which they receive feedback regarding the accuracy of their responses. The experimental task consisted of four blocks, each containing 30 congruent and 30 incongruent trials, presented in randomized order. For each participant, we extracted reaction time metrics for correct responses to congruent and incongruent trials, with the reaction time difference between the two trial types as a measure of inhibitory control efficiency.

Stop signal task. During the stop-signal delay task, participants are instructed to press the right arrow-key when presented with an X and the left arrow-key when presented with an O. These trials are called “go-trials”. On 25% of trials, the X or O are accompanied by a 1000 Hz auditory “stop cue”. When the stop cue is presented, participants are instructed to withhold their motor response to the X or O. These are called “stop-trials”. For both stop- and go-trials, participants were presented with an initial fixation for 500ms, followed by the visual stimuli presented for 1000ms, and lastly, a blank screen was presented for a pseudorandom duration between 600-900ms. For “stop-trials”, the time elapsed between the presentation of the go-stimulus and the stop cue (stop-signal delay; SSD) varies as a function of performance to calibrate successful inhibition of the go-response to 50% of stop-trials. Initially the SSD is set to 250ms and increased by 50 ms following correct responses to stop-trials and decreased by 50 ms following incorrect responses to stop-trials. Performance measures within the current project confirm that the calibration procedures resulted in approximately 50% successful inhibition trials within our samples (Average percent correct responses on stop trials: CFA sample=52.54%, CALM-IT sample=51.63%). Following completion of two 16-trial practice blocks, participants completed 5 experimental blocks with 88 trials each, resulting in a total of 330 go-trials and 110 stop-trials. For each participant, we extracted the stop-signal reaction time (SSRT), the difference between the average SSD and the average reaction time to go-trials, as a measure of inhibitory control.

Table S1. Raw bivariate correlations between CALM-IT d-prime and inhibitory control in-laboratory task performance

	CALM-IT D-prime	
	Levels 1-5	Levels 6-10
SST SSRT	-0.340**	-0.445***
Flanker RT Diff	-0.092	-0.091
AS % Correct	0.283*	0.216
AXCPT d'	0.215†	0.286*

Note. †*p*<.01, **p*<.05, ***p*<.01, ****p*<.001

Text S2. Detail of confirmatory factor analyses

All confirmatory factor analyses were conducted using Mplus (v8.5). Please see the Statistical analyses in the main manuscript for details on the specific models. For all models, fit was assessed using the comparative fit index (CFI), Tucker Lewis index (TLI), and root mean square error of approximation (RMSEA). Strong model fit was indicated by CFI and TLI, values >0.95 and RMSEA <0.05. Full-information maximum likelihood (FIML) was used to address missing data.

Text S3. Details of fMRI data processing and analysis

Analysis of Functional Neuroimages (AFNI; version_23.1.08) and Freesurfer were used for all imaging analyses. First, each participant's anatomical volume was processed using Freesurfer's recon-all to generate whole brain parcellations. AFNI's @SSwarper was used to perform skull stripping and estimate nonlinear warps to standard MNI space. Results from Freesurfer and @SSwarper were included in the afni_proc.py that was used for standard preprocessing, which included removal of the first four TRs, de-spiking, slice time correction and epi-anatomical alignment using the lpc+ZZ cost function.[53] Fast ANATICOR[54] was also included alongside motion censoring of TR pairs with Euclidean norm >1mm or an outlier fraction of >10%. A whole brain mask was derived via intersecting the EPI and anatomical scan. Data was spatially smoothed within the mask to FWHM=6.5 mm (effective smoothness of 9mm) and scaled to the local average to allow interpretation of effect estimates as % signal change.

Individual level general linear models were fitted using 3dREMLfit applying generalized least squares with a temporal autocorrelation structure of ARMA(1,1). Baseline, low frequency drifts and six motion regressors were included per run in addition to six regressors of interest that were time-locked to the stimulus onset using a GAM variate basis function. Task regressors modelled trial condition (congruent or incongruent trials) and response type (correct, commission error, or omission error). Afni_proc.py's QC HTML[55] was used for quality control assessment. Participants with missing data, an accuracy of <70%, average motion per TR of >.25mm after censoring or >15% of TRs censored were excluded from further analyses (*n*=26).

A complementary voxel-wise whole brain analysis was conducted applying the same model using AFNI's 3dMVM.[59] Monte Carlo simulations were performed via AFNI's 3dClustSim to correct for multiple comparisons. All analyses were restricted to a whole-brain mask of 151,684 voxels where 90% of participants contributed data. Smoothness of the residuals was estimated based on a Gaussian plus mono-exponential spatial autocorrelation function (3dFWHMx with -acf flag) for all participants and averaged (ACF parameters, a=0.57, b=3.39, c=9.58). Two-sided thresholding was examined for whole-brain tests with first-nearest neighbor clustering (NN = 1). To obtain a whole-brain family-wise error correction of p < .05, all results were thresholded at a voxel-wise *p* < .001 and a cluster extent of *k* = 26 voxels.

Fig S1. Confirmatory Factor Analysis (CFA) results for a single latent factor of inhibitory control estimated from observed behavior across of the four canonical in-laboratory tasks. Note. Values represent standardized factor loadings. CFI=0.989, TLI=0.967, RMSEA=0.40 CI_{90}[0-0.129].

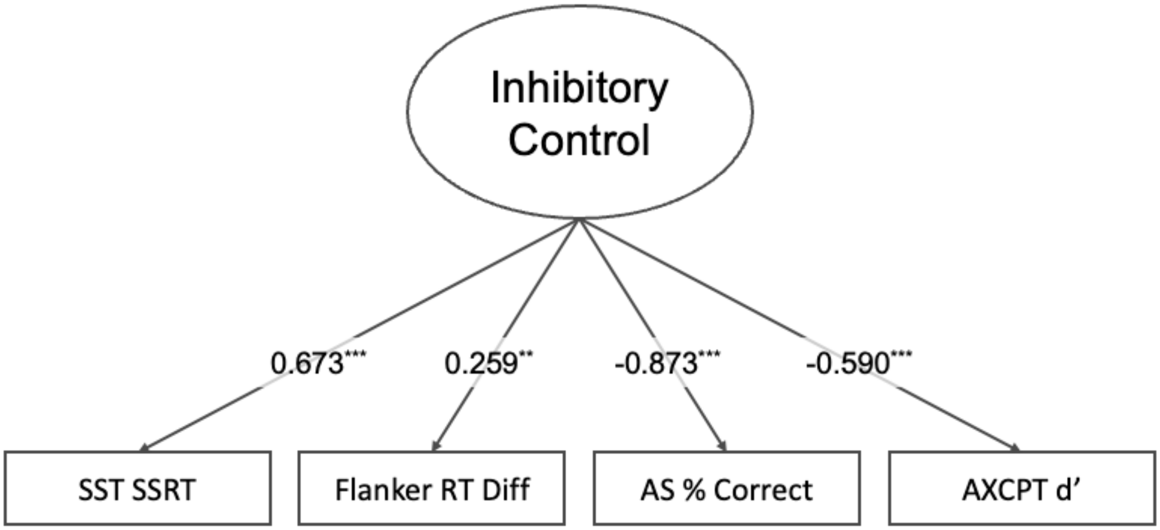

Fig S2. Confirmatory bifactor model of childhood psychopathology Note. Values represent standardized factor loadings. CFI=0.993, TLI=0.991, RMSEA=0.49 CI_{90}[0.029-0.066].

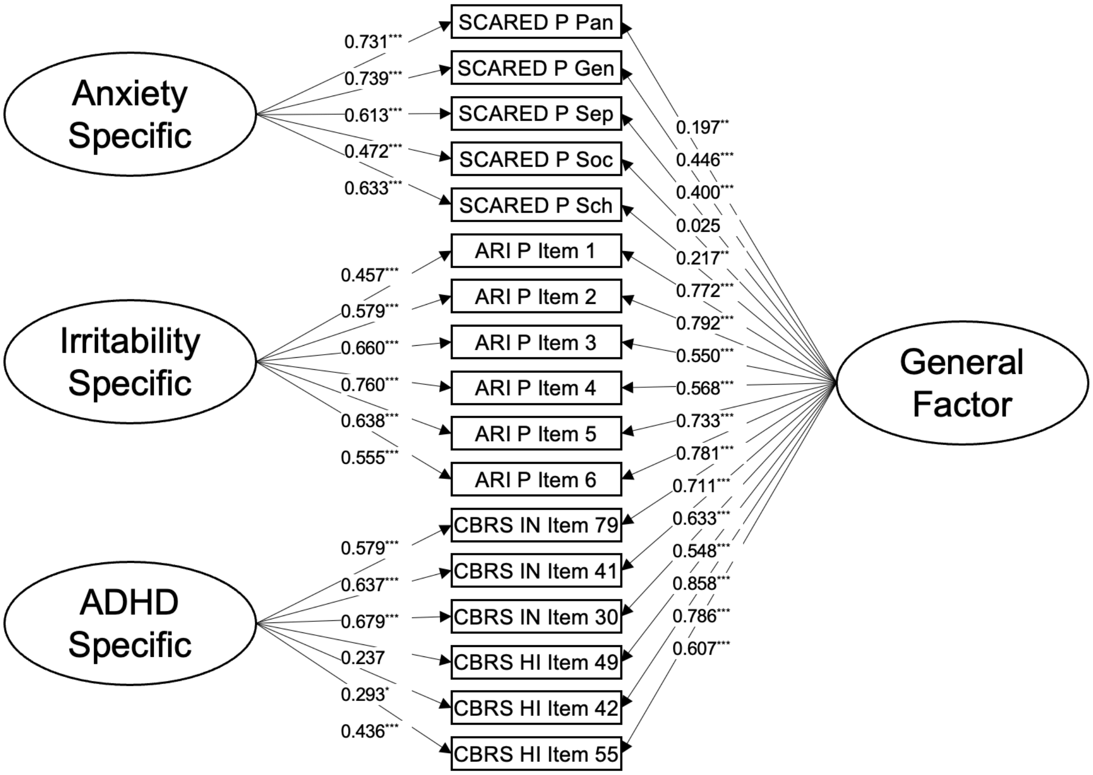

Table S2. Raw bivariate correlations between CALM-IT d-prime and childhood psychopathology

	CALM-IT D-prime	
	Levels 1-5	Levels 6-10
General Factor	**-0.289*** **	**-0.328*** **
Anxiety Specific Factor	0.006	0.102
Irritability Specific Factor	-0.021	0.048
ADHD Specific Factor	0.015	-0.086
SCARED Child Total	-0.064	-0.035
SCARED Parent Total	-0.072	0.005
ARI Child Total	**-0.194***	**-0.176***
ARI Parent Total	**-0.214****	**-0.185***
CBRS DSM IV ADHD Total	**-0.201***	**-0.291***
CBRS DSM IV Inattentive	-0.112	**-0.217***
CBRS DSM IV Hyperactive Impulsive	**-0.271*** **	**-0.330*** **
MFQ Child Total	-0.104	0.014
MFQ Parent Total	-0.057	-0.073

Note. **p<.05, **p<.01, ***p<.001*
