# Supplementary material for: Leveraging technology to probe mechanisms of psychopathology: A proof of concept study of inhibitory control
Source: PLoS One. 2025 Jun 6;20(6):e0319004. doi: 10.1371/journal.pone.0319004 (PMC12253936; doi:10.1371/journal.pone.0319004)
Supplement: Attachment — Submitted filename: PONE-D-24-31796 Response to Reviewers.pdf [file pone.0319004.s003.pdf]

We thank the editor/reviewers for the opportunity to revise our manuscript for consideration at Plos ONE. Our response to each comment can be found below in RED BOLD text.

Journal Requirements:

1. Please ensure that your manuscript meets PLOS ONE's style requirements, including those for file naming. The PLOS ONE style templates can be found at https://journals.plos.org/plosone/s/file?id=wjVg/PLOSOne_formatting_sample_main_body.pdf and https://journals.plos.org/plosone/s/file?id=ba62/PLOSOne_formatting_sample_title_authors_affiliations.pdf

We have updated all of the formatting in the manuscript to reflect PLOS ONE's style requirements.

2. We note that the grant information you provided in the 'Funding Information' and 'Financial Disclosure' sections do not match.

When you resubmit, please ensure that you provide the correct grant numbers for the awards you received for your study in the 'Funding Information' section.

Both the 'Funding Information' and 'Financial Disclosure' sections of the submission state ZIAMH002781 as the funding source. The additional information in the financial disclosure reports the protocols but not additional funding.

3. Thank you for stating the following financial disclosure:
This research was supported by the NIMH Intramural Research Program (ZIAMH002781), conducted under NIH Clinical Study Protocols 15-M-0182 (ClinicalTrials.gov identifier: NCT02531893), 02-M-0021 (NCT00025935), 00-M-0198 (NCT00006177), and 01-M-0192 (NCT00018057).

Please state what role the funders took in the study. If the funders had no role, please state: ""The funders had no role in study design, data collection and analysis, decision to publish, or preparation of the manuscript.""
If this statement is not correct you must amend it as needed.
Please include this amended Role of Funder statement in your cover letter; we will change the online submission form on your behalf.

In our cover letter for our revision submission we now include "This work was supported by the NIMH Intramural Research Program. The funders had no role in study design, data collection and analysis, decision to publish, or preparation of the manuscript."

4. Thank you for stating the following in the Acknowledgments Section of your manuscript:
This research was supported by the NIMH Intramural Research Program (ZIAMH002781), conducted under NIH Clinical Study Protocols 15-M-0182 (ClinicalTrials.gov identifier: NCT02531893), 02-M-0021 (NCT00025935), 00-M-0198 (NCT00006177), and 01-M-0192 (NCT00018057).

We note that you have provided funding information that is not currently declared in your Funding Statement. However, funding information should not appear in the Acknowledgments section or other areas of your manuscript. We will only publish funding information present in the Funding Statement section of the online submission form.

Please remove any funding-related text from the manuscript and let us know how you would like to update your Funding Statement. Currently, your Funding Statement reads as follows:
This research was supported by the NIMH Intramural Research Program (ZIAMH002781), conducted under NIH Clinical Study Protocols 15-M-0182 (ClinicalTrials.gov identifier: NCT02531893), 02-M-0021 (NCT00025935), 00-M-0198 (NCT00006177), and 01-M-0192 (NCT00018057).

Please include your amended statements within your cover letter; we will change the online submission form on your behalf.

We have removed all funding information from the Acknowledgments section and have updated the cover letter.

5. Your abstract cannot contain citations. Please only include citations in the body text of the manuscript, and ensure that they remain in ascending numerical order on first mention.

We have confirmed that no citations have been included in the abstract.

6. We note that Figures 1, 2, and 3 in your submission contain copyrighted images. All PLOS content is published under the Creative Commons Attribution License (CC BY 4.0), which means that the manuscript, images, and Supporting Information files will be freely available online, and any third party is permitted to access, download, copy, distribute, and use these materials in any way, even commercially, with proper attribution. For more information, see our copyright guidelines: http://journals.plos.org/plosone/s/licenses-and-copyright.

We require you to either (1) present written permission from the copyright holder to publish these figures specifically under the CC BY 4.0 license, or (2) remove the figures from your submission:

a. You may seek permission from the original copyright holder of Figures 1, 2, and 3 to publish the content specifically under the CC BY 4.0 license.

We recommend that you contact the original copyright holder with the Content Permission Form

(http://journals.plos.org/plosone/s/file?id=7c09/content-permission-form.pdf) and the following text:
"I request permission for the open-access journal PLOS ONE to publish XXX under the Creative Commons Attribution License (CCAL) CC BY 4.0 (http://creativecommons.org/licenses/by/4.0/). Please be aware that this license allows unrestricted use and distribution, even commercially, by third parties. Please reply and provide explicit written permission to publish XXX under a CC BY license and complete the attached form."

Please upload the completed Content Permission Form or other proof of granted permissions as an ""Other"" file with your submission.

In the figure caption of the copyrighted figure, please include the following text: "Reprinted from [ref] under a CC BY license, with permission from [name of publisher], original copyright [original copyright year]."

b. If you are unable to obtain permission from the original copyright holder to publish these figures under the CC BY 4.0 license or if the copyright holder's requirements are incompatible with the CC BY 4.0 license, please either i) remove the figure or ii) supply a replacement figure that complies with the CC BY 4.0 license. Please check copyright information on all replacement figures and update the figure caption with source information. If applicable, please specify in the figure caption text when a figure is similar but not identical to the original image and is therefore for illustrative purposes only.

All images were created by the research team. We own the rights to all images and have not include any copyrighted images.

7. Please include captions for your Supporting Information files at the end of your manuscript, and update any in-text citations to match accordingly. Please see our Supporting Information guidelines for more information: http://journals.plos.org/plosone/s/supporting-information.

We now include captions for our two supplementary figures in the supporting information section at the end of the manuscript.

Reviewer 1

The submission “Leveraging technology to probe mechanisms of psychopathology: A proof of concept study of inhibitory control“ reports several results from a newly developed game that is proposed to measures inhibitory control. The authors report data on the reliability, convergent validity with laboratory-based measures, associations with mental health symptoms (subclinical ADHD) and fMRI. The authors tested a large sample of adolescents, mostly diagnosed with ADHD or mood/anxiety disorder, or healthy controls. Overall, the submission is very comprehensive and contributes a lot of exciting results. My concern with the submission is that the research dilutes the precision of inhibitory control tasks, and their new app presents a unitary approach that claims to assess inhibitory control. It does not further inform about mechanisms, though, at least in the current shape of the submission – more information about the correlations with individual laboratory tasks, and much more details about the tasks themselves are needed. It should be discussed and made transparent to future users of the app that their measurement is not precisely characterized as inhibitory control in other lab paradigms. Furthermore, the presentation is not very critical and does not (yet) provide a clear description of the scientific contribution.

> **We thank the reviewer for their enthusiasm regarding our investigation and presented results. We address each of their comments below to enhance the clarity and precision of our manuscript.**

1. Was the study preregistered? It says “registered report” under article type, I assume this is a mistake and should be an original report, because I could not find any mention of the stage I submission or the preregistration of their data analyses.

> **The submitted work was published as a registered report protocol at PLOS ONE on June 4th 2021. A link to the registered report protocol was included as part of the submission process. To ease access to the registered report we include a link to the publication below:**
>
> **https://journals.plos.org/plosone/article?id=10.1371/journal.pone.0252245**

2. I could not review whether all data are available, but the authors stated that they would publish all data on OpenNeuro. In my view this is perfectly fine and it will certainly be a great asset for future meta-analytic studies.

> **A reviewer link to our OpenNeuro dataset was included as part of the submission process. To ease access to the dataset we include a link below:**
>
> **https://openneuro.org/crn/reviewer/eyJhbGciOiJIUzI1NiIsInR5cCI6IkpXVCJ9.eyJzdWIiOiI5MDYyMDNmMS1lMTEyLTQ4YzktYmFiZi01NjA2OGUxNGE2MzQiLCJlbWFpbCI6InJldmlld2VyQG9wZW5uZXVyby5vcmciLCJwcm92aWRlciI6Ik9wZW5OZXVybyIsIm5hbWUiOiJBbm9ueW1vdXMgUmV2aWV3ZXIiLCJhZG1pbiI6ZmFsc2UsInNjb3BlIc**

yI6WyJkYXRhc2V0OnJldmlld2VyIl0sImRhdGFzZXQiOiJkczAwNTE2NiIsImlhdCI6MTcxNjMxNDQzMSwiZXhwIjoxNzQ3ODUwNDMxfQ.FDgGQiG7u7LGB9aGS49eOJcbx_PXC2u4LNnAwyf-hPU

3. More precision in the introduction regarding what the innovation of the app is required. What aspect of inhibitory control is measured, and what is the benefit of using the app instead of a brief but precise laboratory assessment, under much better controlled conditions?

We appreciate the opportunity to provide more clarity regarding the benefits of the use of mobile-based gamified assessments of inhibitory control such as CALM-IT. We now include the following in our introduction (ps. 3-4):

> **"We designed CALM-IT to build off the strengths of traditional laboratory-based cognitive control tasks while addressing issues with engagement, accessibility, and cost-effectiveness of these often laborious and time-intensive experimental tasks particularly within developmental samples.[5] To do this, we leveraged knowledge from canonical inhibitory control laboratory tasks and recent advances in the gamification of inhibitory control assessment. By using a game-based interface that can be completed using mobile-devices, CALM-IT was designed to extend traditional laboratory-based cognitive control tasks into an engaging, accessible mobile assessment of inhibitory control. While gamification has proven an effective technique for increases engagement while preserving validity of performance, findings are mixed.[8–10] Therefore, it is critical that these novel apps undergo systematic evaluation. As such, we provide preliminary data demonstrating feasibility, accessibility and preliminary validity of this mobile application.[11]"**

4. The description of the task is missing a lot of detail to understand what participants did. How long were stimuli presented? Which stimuli were presented? In the SST variant, which delays were randomly presented and how often? Are participants instructed not to wait on the task? The consensus guide on SST can be used to check whether all relevant data are reported (Verbruggen et al., 2019, eLife).

All tasks are described in detail in Text S1. We have added additional information about the timing of the stimulus presentation:

> **Antisaccade Task: *"Each trial begins with a preparatory period during which participants are presented with either a green or red instructional fixation cross for either 2 or 6 seconds. The color of the cross indicated the next trial is either a prosaccade (green) or antisaccade (red) trail. This preparatory period was followed by a 200 ms blank screen. Next, during both the prosaccade and antisaccade trials, the response period consisted of a yellow visual target presented for 1 second in a pseudorandomized***

location 630 pixels or 315 pixels to the left or right of the center of the screen."

AX Continuous Performance Task (AXCPT): ***"All trials consisted of the following sequence of events: 200-500 ms jittered fixation, 500 ms presentation of the cue, 1000 ms fixation screen, 3900 ms blank screen, 500 ms probe, and a 1700 ms fixation."***

Flanker Task: ***"Participants are instructed to press the left or right arrow button to indicate the direction of the central arrow in series of five side-by-side arrows centered on the screen. The trial terminates upon response and participants are instructed to respond as quickly as they can. If participants do not respond, the response window closed after 2 seconds. All flanker trials were preceded by a 300ms fixation. Following the participants response, a blank screen was presented for a pseudorandom duration (1000ms – 1500ms)."***

Stop signal task: ***"For both stop- and go-trials, participants were presented with an initial fixation for 500ms, followed by the visual stimuli presented for 1000ms, and lastly, a blank screen was presented for a pseudorandom duration between 600-900ms."***

Given specific concerns regarding the SST task, we now report the average percentage correct stop-trials across both the CFA sample and the CALM-IT sample confirming that performance was as expected (p. X):

"Performance measures within the current project confirm that the calibration procedures resulted in approximately 50% successful inhibition trials within our samples (Average percent correct responses on stop trials: CFA sample=52.54%, CALM-IT sample=51.63%)."

Finally, all details regarding the calibration of the stop signal delays was included in Text S1:

"For "stop-trials", the time elapsed between the presentation of the go-stimulus and the stop cue (stop-signal delay; SSD) varies as a function of performance to calibrate successful inhibition of the go-response to 50% of stop-trials. Initially the SSD is set to 250ms and increased by 50 ms following correct responses to stop-trials and decreased by 50 ms following incorrect responses to stop-trials."

5. The authors reported d-prime, collected from their app, as a measure of inhibitory control. I would like to reflect whether inhibitory control is best defined by taking into considerations all hits, misses, across go-/no-go and stop-signal conditions. It is not clear what their task measures and what the outcome reflects.

We chose to operationalize behavior on the task using d-prime as it most directly assesses our target behavior in the task: the ability to flexibly and accurately engage in motor inhibition. This requires not just withholding responses during inhibition trials but also successfully engaging in the target trials. D-prime is an ideal metric for this as it assesses one's ability to discriminate signal from noise by accounting for both the correct hit rate and the false alarm rate. We now include additional language in the statistical analyses section to explain this further (p. 12):

> ***"As such, CALM-IT d-prime assesses both the ability to successfully engage in target motor responses while simultaneously successfully engaging motor inhibition when required. Examination of either correct hit rate or false alarm rate alone would insufficiently capture our behavioral target. For example, participants who were overly cautious or distracted and withheld swipes for both targets and stars would inaccurately appear to have strong inhibitory control performance if false-alarm trials alone were examined."***

6. Related to the point above, I would like to see the separate correlations with SSRT, false alarms, congruency effect in the Flanker task, and so on, rather than a composite latent score. This will help to better elucidate the cognitive mechanism that is measured.

We have added an additional supplementary table (Table S1) that includes the raw bivariate correlations between CALM-IT d-prime and performance on each of the four inhibitory control tasks.

Table S1. Raw bivariate correlations between CALM-IT d-prime and inhibitory control in-laboratory task performance

	CALM-IT D-prime	
	Levels 1-5	Levels 6-10
SST SSRT	-0.340**	-0.445***
Flanker RT Diff	-0.092	-0.091
AS % Correct	0.283*	0.216
AXCPT d'	0.215†	0.286*

*Note. †p<.01, *p<.05, **p<.01, ***p<.001*

7. The association of age and measures from the tasks is mentioned in the discussion, please also include the results.

We have confirmed that all age-related associations included in the discussion section can also be found in the results section:

Language from Discussion (p. 18): ***"Third, performance related to both a latent variable of inhibitory control from standardized in-clinic tasks and neural activation. However, only associations with neural activation to conflict in the IFG remained significant after controlling for age."***

- **Language from Results (p. 16):** ***"After controlling for age, no associations between CALM-IT d-prime and inhibitory control latent factor scores survived (Levels 1-5 b=0.14 p=0.128, Levels 6-10 b=0.14, p=.133)." and*** **(p. 17):** ***"The right and left IFG showed a condition-by-CALM-IT d-prime score interaction for levels 6-10 (left IFG: F(1,48)=4.88, p=.032; right IFG: F(1,48)=7.68, p=.008), but not levels 1-5 (all ps>.05), such that increased activation in the bilateral IFG in response to conflict was associated with better CALM-IT performance."***

Language from Discussion (p. 19): ***"While associations emerged between CALM-IT inhibitory control behavior and inhibitory control latent factor scores, this association did not remain after controlling for age."***

- **Language from Results (p. 16):** ***"After controlling for age, no associations between CALM-IT d-prime and inhibitory control latent factor scores survived (Levels 1-5 b=0.14 p=0.128, Levels 6-10 b=0.14, p=.133)."***

Language from Discussion (p. 20): ***"Finally, we identified clinical relevance through associations with measures of anxiety, irritability, and ADHD symptoms and found that impaired inhibitory control, as measured by CALM-IT, was associated with higher levels of the general factor (i.e., shared or comorbid psychopathology), even after controlling for age."***

- **Language from Results (p. 17):** ***"Associations between general psychopathology factor scores remained significantly associated with both CALM-IT levels 1-5 d-prime, b=-0.16 p=0.047, and levels 6-10 d-prime, b=-0.22 p=0.006, even after controlling for age." and*** **(p. 18):** ***"After controlling for age, all associations between self- and parent-report symptoms with CALM-IT performance were no longer significant."***

We have also reiterated language from the Statistical analyses section in the results section to clarify that the fMRI analyses control for age(p. 17):

"All analyses controlled for age by including age as between-subjects variable."

8. Roadmap: I wonder whether the authors would agree, in retrospect, to include more early piloting and technical testing (given the reported loss of data due to technical issues). I also wondered whether there was any patient involvement in the development of the app. How does the reliability, and validity, inform the further steps?

We agree with the reviewer that piloting and technical testing are a critical part of all scientific work particularly those that include collaboration with 3rd party technical providers. We conducted months of technical piloting and development prior to participant recruitment. We also conducted feedback meetings with participants while designing the task. Feedback from the youth informed our decisions about colors and the background story. However, some technical difficulties cannot be avoided particularly with human subject research. As such, data loss of 4 participants (2% of the sample) is highly consistent with work done in this area particularly when using more naturalistic data collection methods.

9. A commercial partner was involved in the design of the app. Please state in the conflicts of interest whether the partner was involved in the study design, analysis, or decision to submit for publication.

We will include that our industry partner was not involved in the study design, analysis, or decision to submit for publication.

10. Are the reported reliabilities and validities sufficient to follow up with a broader adaptation of the app? And what is the actual use case? I am not convinced that the app provides a solid basis for treatment decisions in the current state

The goal of this paper was not to provide recommendation of the broader adoption of CALM-IT specifically but to rather present a roadmap for the development of targeted mobile applications. We do not feel comfortable asserting that CALM-IT should be used at this current stage as a basis for treatment decisions nor providing criteria for evaluating a guide for how to assess when these applications would meet sufficient reliability and validity for such use. As such, we conclude the manuscript with the following statement (p. 22):

> ***"This work represents an important first step, and future work is needed examine the potential for targeted mobile applications such as CALM-IT to predict symptom time courses and treatment outcomes."***

11. The effects of the employed gamification elements on inhibitory control assessment should be discussed. There are several studies that reported positive and negative effects on engagement but also on performance, e.g.:

Friehs, M. A., Dechant, M., Vedress, S., Frings, C., & Mandryk, R. L. (2020). Effective gamification of the stop-signal task: two controlled laboratory experiments. JMIR Serious Games, 8(3), e17810.
Schroeder, P. A., Lohmann, J., & Ninaus, M. (2021). Preserved inhibitory control deficits of overweight participants in a gamified stop-signal task: experimental study of validity. JMIR Serious Games, 9(1), e25063.
Lumsden, J., Skinner, A., Coyle, D., Lawrence, N., & Munafo, M. (2017). Attrition from web-based cognitive testing: a repeated measures comparison of gamification techniques. Journal of medical Internet research, 19(11), e395.

We thank the reviewer for this important references and now include the following in our introduction section (ps. 3-4):

"By using a game-based interface that can be completed using mobile-devices, CALM-IT was designed to extend traditional laboratory-based cognitive control tasks into an engaging, accessible mobile assessment of inhibitory control. While gamification has proven an effective technique for increases engagement while preserving validity of performance, findings are mixed.[8–10] Therefore, it is critical that these novel apps undergo systematic evaluation."

Reviewer 2

1. Second hypothesis: Why is accuracy conceptualized as an index of task validity rather than an index of the capacities and skills of users?

> **We believe the reviewer is referring to the examination of task performance as an index of feasibility and engagement. If so, the reviewer is correct that the goal of these feasibility analyses was to assess the alignment of the task design with the capacities and skills of our target users with these performance estimates showing that the task is appropriately designed for use in clinical pediatric settings. We now state this in the discussion (p. 19):**
>
> > ***"Next, we demonstrated feasibility and engagement with CALM-IT. Youth and families overwhelmingly exhibited interest in participating in the protocol and once enrolled, participants had high completion rates and performance accuracy confirming that the task design aligns with the developmental capacities and skills of clinical pediatric samples."***

2. CALM-IT: how do stard become a prepotent signal as in traditional go/no-go tasks, where the inhibition is inhibition of a prepotent response?

> **Identical to classic go/no-go tasks that use visual discrimination to differentiate between go and no-go stimuli, CALM-IT was designed such that go stimuli and stop stimuli engage the same motoric response (swiping) but differ only in the nature of the visual cue (color of the space object). Since the no-go stimuli appear less frequently, the motor response to the go-trials quickly becomes prepotent and requires inhibition during the less frequent no-go trials. Thus, the prepotent response is swiping to all stimuli; however, one has to withhold that prepotent response when the yellow "star" is presented.**

3. More information is needed on statistical tests: please list all software and versions that were used for each analysis. please, list all relevant assumptions and how those were tested. ICCs are also insufficiently described, e.g. was absolute agreement assessed?

> **We now include specification of each software and version for our analyses**
>
> > **(p. 13): "*Test-retest reliability of CALM-IT task performance was analyzed in IBM SPSS Statistics (v28) using intraclass correlation coefficients (ICC)"***
> >
> > **(p. 13): "*Using measured variables from each of the four laboratory tasks, a latent factor of inhibitory control was estimated using confirmatory factor analyses conducted in Mplus (v8.5)."***

(p. 14): "*Analysis of Functional Neuroimages (AFNI; version_23.1.08)*[51] *and Freesurfer*[52] *were used for all imaging analyses."*

(p. 14): "*Consistent with prior work*[36]*, shared and unique variances of irritability, anxiety, and ADHD symptoms were estimated using a bifactor model conducted in Mplus (v8.5)"*

(p. 15): "*Associations between clinical symptoms and CALM-IT inhibitory control performance was examined using bivariate correlations in IBM SPSS Statistics (v28)."*

Regarding concerns of our ICC analyses, we have reported the analyses that were approved in our analytic plan from the registered report protocol for this work: "Because we are investigating test-retest reliability, or the stability across measurement, we will use ICC(3,1) which is a two-way mixed effects assessment of consistency within a single measurement. An ICC value of less than 0.40 would indicate poor consistency, an ICC value of 0.40–0.70 would indicate moderate consistency, an ICC value of 0.70–0.90 would indicate good consistency, and an ICC value greater than 0.90 would indicate excellent consistency." We chose to pursue this analytic approach rather than absolute agreement as we expect that inhibitory control behaviors may naturally fluctuate when measure as a momentary construct as such, we are less concerned on the exact performance score being identical across time. Absolute agreement is a more appropriate measure for assessing consistency of reports for example across different respondents (ie. two separate clinicians) when validating assessment tools. As a result, we have chosen to keep the ICC results as reported in the manuscript.

4. More detail should also be provided on the CFA. Provide references for the cutoffs used.

We have added supplementary text to provide additional information regarding the CFAs including the cutoffs used.

Text S2. Detail of confirmatory factor analyses

All confirmatory factor analyses were conducted using Mplus (v8.5). Please see the Statistical analyses in the main manuscript for details on the specific models. For all models, fit was assessed using the comparative fit index (CFI), Tucker Lewis index (TLI), and root mean square error of approximation (RMSEA). Strong model fit was indicated by CFI and TLI, values >0.95 and RMSEA <0.05. Full-information maximum likelihood (FIML) was used to address missing data.

5. it is unclear why the results state "). After controlling for age, all associations between self- and parent-report symptoms with CALM-IT performance were no longer significant." but the discussion and abstract treats this findings as remaining significant after accounting for age.

> **We believe the section of the discussion that the reviewer is referring to regards the findings between the *latent* factor for general psychopathology as estimated by the bifactor model. We have added language to clarify and the section now reads as follows (p. 20):**
>
> > ***"Finally, we identified clinical relevance through associations with measures of anxiety, irritability, and ADHD symptoms and found that impaired inhibitory control, as measured by CALM-IT, was associated with higher levels of the general latent factor for our bifactor model of clinical symptoms (i.e., shared or comorbid psychopathology), even after controlling for age."***

6. it does not appear that - given the novelty of the research question - region of interest analyses are warranted. ROI analyses introduce pre-selection bias and have less generalizability and limited scope. there is not enough precedent to justify a ROI approach.

> **We understand concerns regarding ROI approaches which was why we additionally include a complementary whole-brain analysis which we report on in our results section (p. 17):**
>
> > ***"A complementary whole-brain analysis did not yield any significant clusters at an appropriately stringent cluster correction (Text S3)."***
